# Influence of Glutaraldehyde Cross-Linking Modes on the Recyclability of Immobilized Lipase B from *Candida antarctica* for Transesterification of Soy Bean Oil

**DOI:** 10.3390/molecules23092230

**Published:** 2018-09-02

**Authors:** Iago A. Modenez, Diego E. Sastre, Fernando C. Moraes, Caterina G. C. Marques Netto

**Affiliations:** 1 Laboratório de Metaloenzimas e Biomiméticos, Departamento de Química, UFSCar, São Carlos-SP 13565-905, Brazil; iago_modenez@hotmail.com; 2 Departamento de Física e Ciências Interdisciplinares, Instituto de Física, Universidade de São Paulo, São Carlos-SP 13563-120, Brazil; diegosastre82@gmail.com; 3 Laboratório de Analítica, Bioanalítica, Biossensores, Eletroanalítica e Sensores, Departamento de Química, UFSCar, São Carlos-SP 13565-905, Brazil; fcmoraes@ufscar.br

**Keywords:** lipase, enzyme immobilization, magnetic nanoparticles, cross-linking types, biodiesel synthesis

## Abstract

Lipase B from *Candida antarctica* (CAL-B) is largely employed as a biocatalyst for hydrolysis, esterification, and transesterification reactions. CAL-B is a good model enzyme to study factors affecting the enzymatic structure, activity and/or stability after an immobilization process. In this study, we analyzed the immobilization of CAL-B enzyme on different magnetic nanoparticles, synthesized by the coprecipitation method inside inverse micelles made of zwitterionic surfactants, with distinct carbon chain length: 4 (ImS4), 10 (ImS10) and 18 (ImS18) carbons. Magnetic nanoparticles ImS4 and ImS10 were shown to cross-link to CAL-B enzyme via a Michael-type addition, whereas particles with ImS18 were bond via pyridine formation after glutaraldehyde cross-coupling. Interestingly, the Michael-type cross-linking generated less stable immobilized CAL-B, revealing the influence of a cross-linking mode on the resulting biocatalyst behavior. Curiously, a direct correlation between nanoparticle agglomerate sizes and CAL-B enzyme reuse stability was observed. Moreover, free CAL-B enzyme was not able to catalyze transesterification due to the high methanol concentration; however, the immobilized CAL-B enzyme reached yields from 79.7 to 90% at the same conditions. In addition, the transesterification of lipids isolated from oleaginous yeasts achieved 89% yield, which confirmed the potential of immobilized CAL-B enzyme in microbial production of biodiesel.

## 1. Introduction

Enzyme immobilization is largely known as an empiric technique to obtain more stable and active enzymes [1]. It is known that few modifications on the enzyme structure, types of supports and immobilization methodology will lead to different outcomes [2,3], which explains the vast literature in this area [4,5]. Among the several available supports for enzyme immobilization, magnetic nanoparticles made of magnetite (Fe_3_O_4_) are particularly interesting due to the large global surface area, high mobility and a strong magnetization effect, which allows them to be recovered and recycled by applying an external magnetic field [6,7]. The single magnetic domains from magnetite nanoparticles are randomly oriented at room temperature, but rapidly align their magnetic moments under the influence of a magnetic field, leading to a strong magnetization and no hysteresis [8]. Moreover, the magnetic field has been described to enhance enzymatic activities and stabilities and its use is still very limited [9]. Moreover, despite of the studies describing enzyme immobilization on magnetic nanoparticles, little is known on how the enzyme structure is affected when it is immobilized on different support types and sizes, and even on how the cross-linking agents affect the binding between enzyme and support [10], modifying the enzymatic catalysis. 

Lipases are well-known versatile enzymes, with numerous uses in biotechnology, organic synthesis, medicinal chemistry, in the food industry, and for biofuel synthesis. A classic example of a stable and highly employed enzyme is lipase B from *Candida antarctica* (CAL-B), which turns CAL-B into a highly desirable enzyme to study the influence of an immobilization protocol on the enzyme activity and stability. The CAL-B enzyme has also the advantage of being able to perform a wide variety of reactions, such as hydrolysis, transesterification, and aldol reactions. This enzyme has also been used for biofuel synthesis, but since CAL-B has the drawback of being inactivated by methanol due to the replacement of water molecules to alcohol in the protein surface [11], immobilization protocols were developed to improve its stability in presence of methanol [12,13]. However, the enzyme stability was not evaluated against protocol changes, since subtle variations on the immobilization methods revealed distinct susceptibility towards methanol [13].

Surface activation is normally required for enzyme immobilization. For instance, magnetite nanoparticles suffer a silanization process carried out by (3-aminopropyl)triethoxylsilane (APTES) coupling agent [14]. The silanization procedure enables the addition of functional groups on the particle surface and protects its magnetic core from oxidation processes due to the contact of iron (2+) to the atmospheric oxygen [15]. Additionally, silanization facilitates the cross-linking between support and enzyme, enabling an easy and direct covalent immobilization. Undoubtedly one of the most applied cross-linking agents is glutaraldehyde [16], but its relatively simple chemical structure can be deceiving, since in aqueous solution it possess several monomeric and polymeric forms in equilibrium [17]. In the protein immobilization context, glutaraldehyde can react with several functional groups such as amine, thiol, imidazole and a variety of amino acids (e.g., lysine, arginine, cysteine and serine), despite of that, it is well known that this reaction tends to occur, mainly, in the 𝜺-amino group of lysyl residues due to its higher nucleophilicity [18]. The 𝜺-amino group nucleophilic attack towards the glutaraldehyde molecule, usually, leads to the formation of Schiff bases. However, the linkage of glutaraldehyde with amino groups showed exceptional stability at extreme pHs and temperatures [10], which does not match to the Schiff base properties [17]. Hence, several alternative mechanisms had been proposed for glutaraldehyde crosslinking, such as a Michael-type addition involving the conjugate addition of the protein amino groups to the α,β-unsaturated oligomers ethylenic double bonds of glutaraldehyde [19]. A slightly different mechanism involved the formation of quaternary pyridinium compounds due to its dimerization in the presence of amino groups [20]. Despite of all reported mechanisms, the reaction of glutaraldehyde with proteins is still under debate. 

**Scheme 1:**
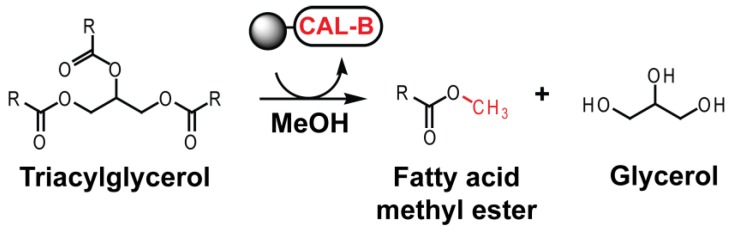
Immobilized CAL-B catalyzed transesterification reaction of triacylglycerol from soy bean oil in methanol to obtain fatty acid methyl ester (FAME also called “biodiesel”).

Recently, Yi et al. described the immobilization of a lipase from *Candida rugosa* on the surface of magnetic nanoparticles inside reverse micelles, and showed an enhanced activity and stability when compared to a nonmicellar immobilization [21]. Therefore, we attempt to analyze the influence of different reverse micelle–made magnetic nanoparticles and glutaraldehyde cross-linking on the activity and stability of CAL-B for oil transesterification reaction in methanol (Scheme 1). Hence, we report the immobilization of this enzyme on three different magnetic nanoparticles synthesized inside reverse micelles by the coprecipitation method and evaluated their transesterification potential of soy bean oil and lipid extracts from oleaginous fungus. Despite no direct correlation of the surfactant size was observed to influence CAL-B activity/stability, it was found a correlation between the nanoparticle agglomerate sizes and the enzymatic stability. Moreover, the glutaraldehyde cross-linking mode influenced the recyclability of immobilized CAL-B, in which Michael-type addition resulted in less stable enzymes.

## 2. Results and Discussion

### 2.1. Characterization of the Magnetic Nanoparticles 

**Figure 1:**
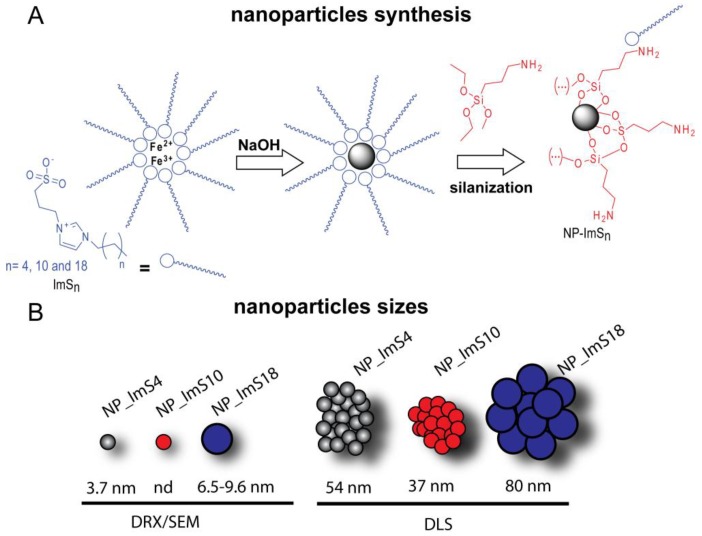
(**A**) Scheme of NP-ImSn synthesis using reverse micelles made of zwitterionic surfactants ImSn (n = 4, 10 and 18); (**B**) Typical sizes obtained for the particles using different analyses.

Three different magnetic nanoparticles were synthesized (NP-ImS4, NP-ImS10 and NP-ImS18) by using inverse micelles, based on Souza et al. protocol [22] in a chloroform/water suspension, containing zwitterionic surfactants with carbon chains of four, ten and eighteen carbons (Figure 1A, Figures S1–S3).

X-ray powder diffraction (XRD) analysis of all synthesized nanoparticles (Figures S4–S6) indicated that the magnetic core was composed of small grains of magnetite with low crystallinity, as was also observed by Salviano et al. [23]. The nanoparticles crystal sizes were determined by using Scherrer equation (Equation (1)), in which *B* is the peak width; *K* is the Scherrer constant (0.94 for spherical crystals); λ is the X-ray wavelength and *L* is the crystallite size. Hence, by XRD analysis, the NP-ImS4 size was approximately of 3.68 nm, whereas for NP-ImS18, the particle size was approximately 6.52 nm. NP-ImS10 could not have their size determined, due to their apparent low crystallinity.

(1) $$\;B\;\left(2\theta \;\right)=\frac{K\lambda }{L\;cos\theta }$$

Due to resolution issues, scanning electron microscopy (SEM) of these nanoparticles only revealed the mean size of NP-ImS18 particles (9.6 nm), since NP-ImS4 and NP-ImS10 were smaller than NP-ImS18 (Figures S7–S9). However, the dynamic light scattering (DLS) measurements of the silanized particles at pH 7 indicated a uniform nanoparticle agglomeration since NP-ImS4, NP-ImS10 and NP-ImS18 (Figures S10–S12) showed a mean diameter of 54, 37, and 80 nm, respectively. In fact, the agglomerated nanoparticles (corresponding to 8–12 units in the case of NP-ImS18) were used for the immobilization protocol (Figure 1B). The agglomeration of nanoparticles might be due to different concentrations used for each analyses, since for SEM a 0.5 mg·L^−1^ was employed, whereas a suspension of 1g·L^−1^ concentration was used for DLS analysis [24]. Following this premise, if a much higher concentration (10g·L^−1^) is employed for DLS analysis, micron-sized agglomerates are observed (Figure S13). 

In addition, infrared analysis of the magnetic nanoparticles after silanization indicated the presence of residual zwitterionic surfactant on the particles surface even after several washing procedures (Figure S14). Microanalysis assays revealed a 4%, 0.6% and 1.5% of surfactant impurity on the surface of NP-ImS4, NP-ImS10 and NP-ImS18 particles, respectively and it was already shown that the presence of detergents might increase lipases activity upon immobilization procedures [25].

### 2.2. Improvement of CAL-B Immobilization Protocol

The immobilization protocol was optimized by finding the best enzyme/nanoparticles ratio, glutaraldehyde concentration and immobilization time. The optimization was based on the yield of soy bean oil transesterification reaction, Scheme 1, which was verified by ^1^H-NMR analyses. For instance, the areas corresponding to the singlet of methoxyl groups from the methyl esters at 3.7 ppm (A1) (Figure S14) and the triplet of the carbonyl methylene groups at 2.3 ppm (A2) (Figure S15) were correlated using Equation (2).

(2) $$\;Y\;(\%)\;=100.\frac{2A1}{3A2}$$

**Figure 2:**
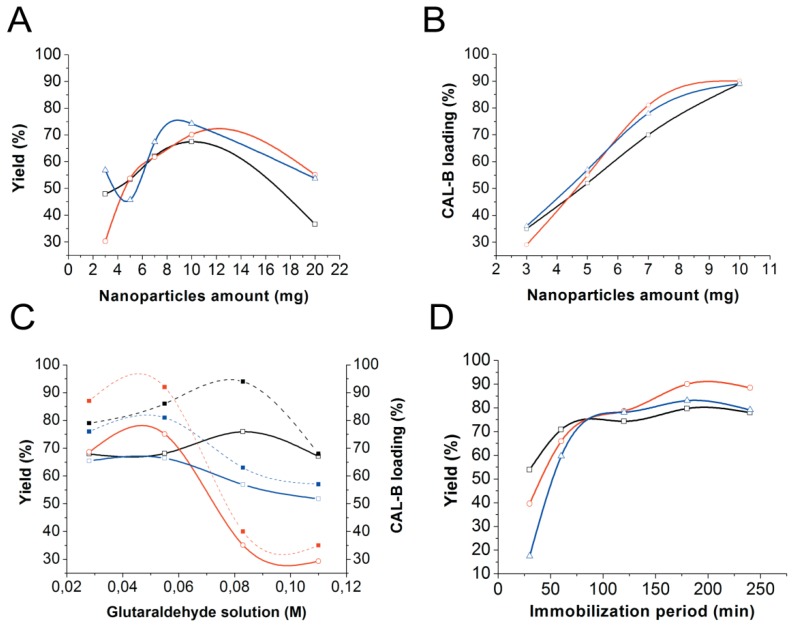
Analysis of the parameters evaluated in the immobilization protocol. (**A**) Effect of the amount of nanoparticles used; (**B**) Load of immobilized CAL-B on the amount of magnetic nanoparticles used; (**C**) Effect of the glutaraldehyde concentration used in the reaction yield (left axis) and in CAL-B loading (right axis- dotted lines) and (**D**) effect of the immobilization time. NP-ImS4/CAL-B (black line, □), NP-ImS10/CAL-B (red line, ○) and NP-ImS18/CAL-B (blue line, △).

Firstly, the amount of magnetic nanoparticles used in the immobilization protocol was evaluated by keeping constant the lipase (10 mg·mL^−1^) and glutaraldehyde (0.028 mol·L^−1^) concentrations, as well as the immobilization period of 60 min. As observed in Figure 2B, higher concentrations of nanoparticles naturally lead to a higher lipase loading, which never achieved 100%, possibly be due to the quantification method used (Bradford method). Classical Bradford method for CAL-B quantification can mistakenly minimize the amount of immobilized CAL-B up to 10 times, as was observed by Nicolás et al. [26]. Hence, possibly, at 10 mg of magnetic nanoparticles used, 100% of CAL-B was already immobilized, which was associated to higher reaction yields (Figure 2A). The increase in yield upon increasing amounts of support reached a maximum at 10 mg of magnetic support, which is probably due to steric hindering effects on the immobilized enzyme.

Different concentrations of the cross-linker were also evaluated (Figure 2C), keeping the lipase concentration (10 mg·mL^−1^) and immobilization time (60 min) constants and using the best nanoparticle amount previously determined (10 mg). The reaction yield was enhanced up to a maximum of 76% for NP-ImS4/CAL-B when 0.083 mol. L^−1^ of glutaraldehyde was used, whereas particles NP-ImS10/CAL-B and NP-ImS18/CAL-B had their maximum conversions of 75 and 66%, respectively, at lower concentrations of glutaraldehyde (0.055 mol. L^−1^). It was seen that excess of glutraldehyde led to the diminishment of CAL-B immobilization, possibly due to intra-CAL-B crosslinking in detriment of immobilization (Figure 2C, dotted lines). However, it was described that high concentrations of cross-linking agent increased the enzyme rigidity, which might have influenced in the active site mobility, leading to a decrease in the reaction yield [27]. Moreover, excessive glutaraldehyde concentrations might affect the tridimensional structure of the enzyme [28].

The immobilization period was also evaluated due to its direct influence on the lipase loading and possibly on the enzyme conformation upon immobilization, since different researchers related a maximum activity in a specific period of time with decrease of the enzyme activity after this period eg [29,30,31,32,33,34]. Some of the reasons for such behavior are possibly: the change of enzyme conformation and denaturation [29], the formation of new bonds between enzyme and support [35], mass transfer problems [36], extensive aggregation of the support [37] or due to steric hindrance against immobilized enzymes [38]. Hence, the amelioration of immobilization time was obtained employing 10 mg of magnetic nanoparticles and 0.083 mol. L^−1^ of glutaraldehyde for NP-ImS4 and 0.055 mol·L^−1^ of glutaraldehyde for NP-ImS10 and NPImS18. In Figure 2D it is seen that the immobilization period has a deep influence on the transesterification yield of all the studied systems. Naturally, longer periods of immobilization results in higher lipase loading on the nanoparticles, in which 180 min was the optimum immobilization time, achieving 79.7, 90 and 83% yield for NP-ImS4/CAL-B, NP-ImS10/CAL-B and NP-ImS18/CAL-B, respectively. Longer immobilization times resulted in the stabilization of the value of transesterification yield (Figure 2D).

### 2.3. Immobilized CAL-B Characterization

**Figure 3:**
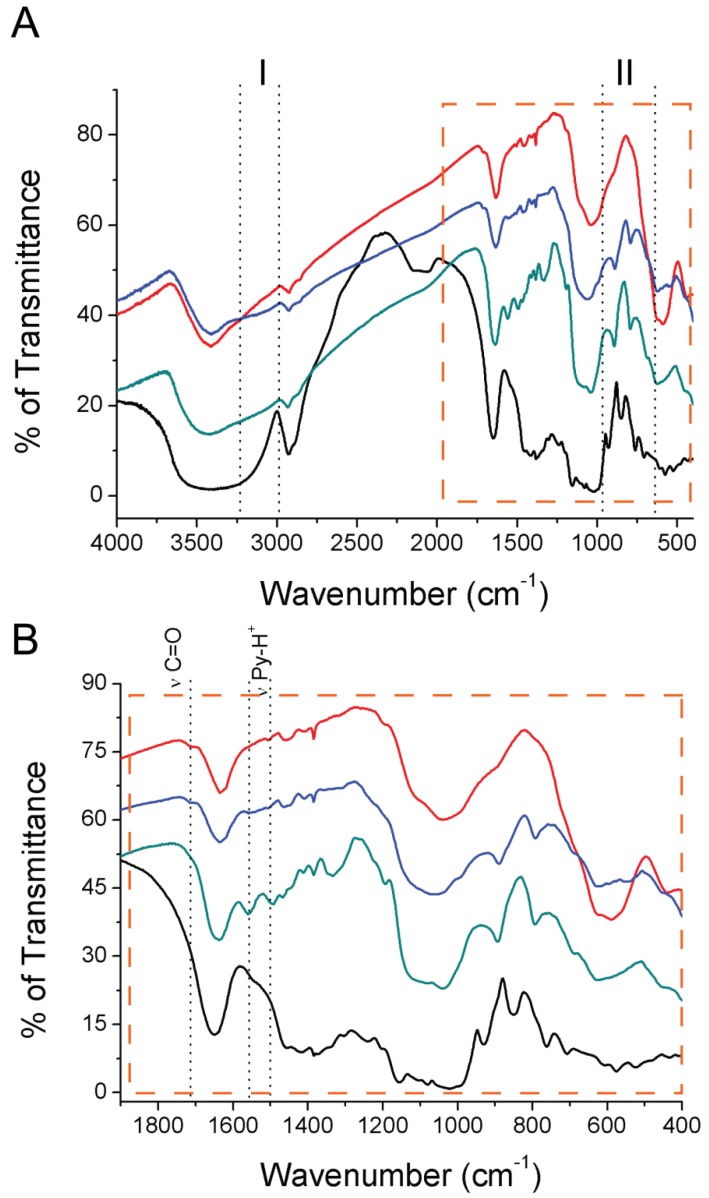
Infrared spectra of free CAL-B (black line), NP-ImS_4_/CAL-B (red line), NP-ImS_10_/CAL-B (blue line), NP-ImS_18_/CAL-B (green line). (**A**) Region from 4000 cm^−1^ to 400 cm^−1^ and (**B)** region from 1850 cm^−1^ to 400 cm^−1^.

After achieving the best immobilization protocol for each of the supports, an infrared analysis was performed in order to provide some insights into CAL-B structure. Interestingly, NP-ImS4/CAL-B and NP-ImS10/CAL-B presented bands at 1715 cm^−1^ in the infrared spectra (Figure 3), which is the region for carbonyl stretching [39], whereas NP-ImS18/CAL-B presented strong bands at 1560 and 1494 cm^−1^ instead, which could be from heterocyclic ring stretching modes [40]. Even though the carbonyl stretching region might include aldehyde, acids, amides, esters, and ketones, it should be noted that Monsan et al. observed a similar band when cross-linked lysine to α,β-unsaturaded polyglutaraldehyde, via a Michael-type addition. In their study, control reactions using crotonaldehyde and lysine also presented a band a 1720 cm^−1^, corroborating to their hypothesis. Moreover, they observed similar stabilities for the glutaraldehyde–lysine bond formed both at pH 7 and 10.3, which would result from the same cross-linking modes formation [41]. Hence, based on this previous study, we attribute the band at 1715 cm^−1^ (Figure 3) to an aldehyde moiety of the cross-link.

It should be noted that NP-ImS10/CAL-B also present the bands at 1560 and 1494 cm^−1^ to some extent, indicating that it is possibly an intermediate between NP-ImS4/CAL-B and NP-ImS18/CAL-B systems. Moreover, although it is not possible to exclude the formation of an imine bond upon cross-linking, due to the presence of a band at 1632 cm^−1^ (Figure 3) before CAL-B immobilization, it is possible to infer that other glutaraldehyde cross-linking structures were also present in the NP/CAL-B systems.

As previously stated, glutaraldehyde has several equilibrium structures in solution, which might interfere on the cross-linking mode for enzyme immobilization [42]. For example, the formation of a quaternary pyridinium moiety has been described when approximately 3000 equivalents of glutaraldehyde were added to an ovalbumin solution at pH 4.2 [20]. Even though a neutral pH and 30 times less excess of glutaraldehyde were employed in our experiments, in order to analyze if the pH is relevant to the cross-linking mode, we performed CAL-B immobilization on NP-ImS18 at three different pHs (4, 7 and 11) followed by FTIR assays (Figure S17). We observed that at pH 4 as well as pH 7, two bands centered at 1560 cm^−1^ and 1413 cm^−1^ were evident, probably from the formation of a pyridinium bond, whereas the aldehyde band (1715 cm^−1^) was more pronounced at pH 11 than at neutral pH. Hence, noticing that our immobilization conditions are different from the one described to form pyridinium, possibly, the zwitterionic surfactants might affect on the local pH of the nanoparticles as was previously described [43,44], in which ImS18 would have a larger impact than ImS10 and ImS4. The local pH change would influence on the formation of a pyridinium/hetorocyclic moiety, and as a proof of concept, we performed CAL-B immobilization on NP-ImS4 in the presence of surfactant ImS18 as a contaminant and surprisingly, the band at 1715 cm^−1^ was not observed (Figure S18), indicating a possible effect of ImS18 on the local pH of the nanoparticle surface. On the other hand, a Michael-type addition [19] was achieved when amine was in a concentration equal to or higher than 2:1 per glutaraldehyde [45]. Moreover, an arginine-lysine cyclic structure was observed when 0.5 mM glutaraldehyde was employed at pH 7.5 [46,47,48]. When considering these facts, NP-ImS4/CAL-B and NP-ImS10/CAL-B systems are possibly cross-linked through a Michael addition, whereas NP-ImS18/CAL-B is possibly cross-linked through the formation of a pyridine ring. The presence of an aldehydic band was also observed in other glutaraldehyde-lipase studies after surface activation [49], and in our FTIR spectra, this band could also be due to the presence of unreacted aldehydic groups. Although, we believe it would be more plausible to come from the cross-linking mode, since the immobilization took long periods to reach completion (up to 2 h) and that glutaraldehyde cross-linking normally occur rapidly under standard conditions [50].

It should be noted that of C–N–H in amides, scissoring/bending of N-H in amines/imines and stretching of C=C in arenes are observed around 1480–1550 cm^−1^, however, NP-ImS10/CAL-B and NP-ImS18/CAL-B systems present other bands that might also be related to pyridine formation, such as the ones located at 3200–3000 cm^−1^ (Figure 3B, region I), 895 cm^−1^, 794 cm^−1^ and 689 cm^−1^ [51] (Figure 3B, region II), again confirming that NP-ImS10/CAL-B system has similar bands to NP-ImS4/CAL-B and NP-ImS18/CAL-B systems. 

**Figure 4:**
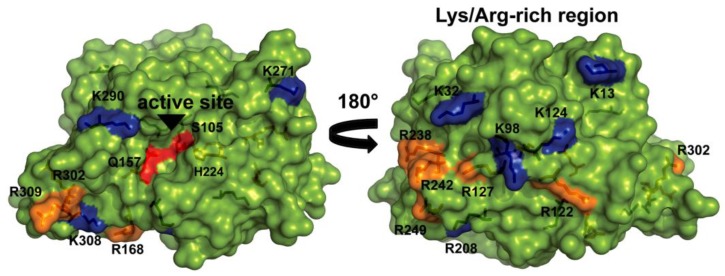
Surface representation of the crystal structure of CAL-B enzyme from *Candida antartica* (PDB ID: 4K6G) showing the location of the exposed lysines (in blue)/arginines (in orange) residues and the catalytic site (in red).

As a matter of fact, our immobilization protocol initiates with the activation of the surface of the magnetic nanoparticles with glutaraldehyde prior to CAL-B addition. Considering that CAL-B has a higher concentration of amine groups at the opposite direction from the active site (Figure 4) [50], we assume that the immobilization took place at this region, letting the active site far away from steric hindrances from the particle. Interestingly, this region is rich on lysines and arginines (Figure 3), two of the most reactive aminoacids towards aldehyde [47,52].

In order to verify the effects of the immobilization on CAL-B transesterification reaction, a comparison based on the energy and entropy of activation was performed. Free CAL-B enzyme showed very low rate constants for each of the reactions temperatures (27 °C, 32 °C and 37 °C) and consequently, the reaction energy of activation (Ea) was the highest among all the samples (29.4 kJ·mol^−1^). These results implicate on the inactive behavior of CAL-B free enzyme on the reaction conditions, with yields around 2% (Table 1). However, an increase up to 45 times on the reaction yield was observed upon CAL-B immobilization, which was accompanied to an increase in the reaction constant and on the decrease on the activation energy. It is important to notice that free CAL-B in presence of ImS10 surfactant did not present any activation behavior (reaction yield: 1.5%, Table 1), indicating that CAL-B activation was due to the immobilization. The observed increase on the activation entropy of immobilized CAL-B might be related to a more mobile enzyme on the transition state, which seemed to aid on the enhancement of the reaction yield. 

### 2.4. Activity and Reuse Stability of Immobilized CAL-B for Biodiesel Synthesis

**Figure 5:**
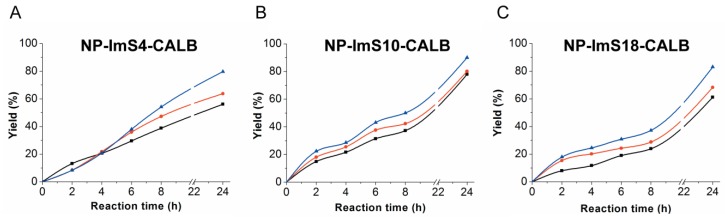
Influence of the transesterification reaction time on the yield of FAMEs production at different temperatures: 27 °C (black line), 32 °C (red line) and 37 °C (blue line) for each of the immobilized systems: (**A**) NP-ImS4-CALB, (**B**) NP-ImS10-CAL-B and (**C**) NP-ImS18-CALB.

The transesterification reaction by immobilized CAL-B was performed at different reaction times (0, 2, 4, 6, 8 and 24 h) and temperatures (27 °C, 32 °C and 37 °C) for a proper evaluation of each immobilized system. As shown in Figure 5, the increase on reaction time and temperature resulted in higher yields for all of immobilized CAL-B, achieving the better transesterification yield (around 80–90%) at 37 °C after 24 h of reaction.

**Figure 6:**
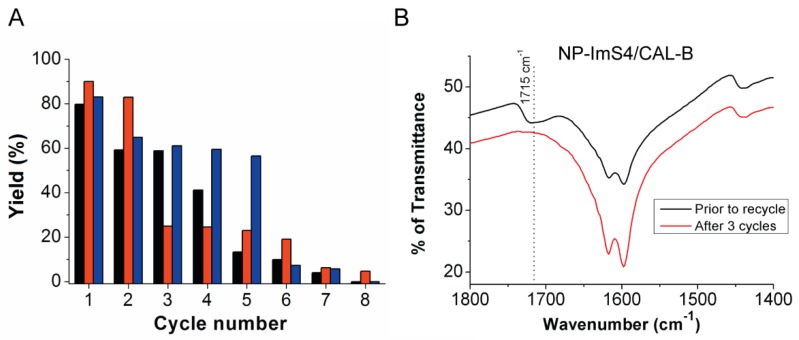
(**A**) Recycling of immobilized CAL-B for transterification reaction. The reaction percentage yield for each system is represented in colored bars: NP-ImS4/CAL-B (black bars), NP-ImS10/CAL-B (red bars) and NP-ImS18/CAL-B (blue bars). (**B**) Infrared spectra of NP-ImS4/CAL-B before (black line) and after (red line) the three reaction cycles.

The stability of immobilized CAL-B was evaluated for its reuse ability (Figure 6). Interestingly, the NP-ImS10/CAL-B system presented the highest catalytic conversion which might be associated to its lower activation energy (Ea = 18.2 KJ·mol^−1^), since lower Ea leads to higher rate constants (*k*) and consequently, on higher turnover frequency of a catalyst [53]. However, the NP-ImS18/CAL-B system was the most stable one, being able to retain 68% of its original activity up to five cycles (Figure 5), whereas NP-ImS4/CAL-B system retained 51% of its original activity up to four cycles and NP-ImS10/CAL-B was the least stable (28% of activity retention up to the third cycle). Surprisingly, the highest stability of NP-ImS18/CAL-B, seems to be associated to a higher activation energy [54] and lower *k* value, combined to the higher entropy of activation than free CAL-B (Table 1). A large negative entropy of activation might be due to the ordering of solvent molecules in the transition state [55], but also from protein surface mobility [56]. Therefore, although the transesterification reaction with NP-ImS18/CAL-B seems to undergo slower (Table 1 and Figure 5) than the other systems; as the *k* value indicates, the highest entropy of activation and more mobile enzymatic surface seems to not interfere on the active site conformation during successive cycles. 

More importantly, NP-ImS18/CAL-B system was the only immobilized system which presented strong pyridine stretching bands in the infrared spectrum (Figure 3). Hence, we assume that the Michael-type cross-linking between CAL-B and NP-ImS4 and NP-ImS10 resulted in the formation of free aldehyde. Michael-type addition reaction involves the nucleophilic attack of the amine (or other nucleophile) to an α,β-unsaturated glutaraldehyde molecule, generating an aminated glutaraldehyde, in which the aldehyde portion remains unreacted. Thus, the presence of free aldehyde on the particle surface might not interfere at the first reaction cycle, but would be able to keep reacting with free amine groups from CAL-B, unfolding it or hindering its active site, which inhibits the catalytic reaction on the successive cycles. In order to verify this possibility, we performed an infrared assay of NP-ImS4/CAL-B before and after three transesterification cycles (Figure 6B) and it was evident that the aldehyde band located at 1715 cm^−1^ vanished after the recycle, corroborating to the hypothesis of a Michael-type addition occurring in NP-ImS4 system. Moreover, other cross-linking reactions, that does not generate aldehydic portions, have been described to produce a high methanol tolerance [57]. 

In our experiment, it was also evident that the largest agglomerates of nanoparticles measured prior to the immobilization protocol (by DLS experiments) had the highest enzymatic stability, suggesting a direct correlation between nanoparticle sizes (agglomerates) and stability, in which NPImS18/CAL-B (agglomerate size = 80 nm; 5 reuse cycles) > NPImS4/CAL-B (agglomerate size = 54 nm; three reuse cycles) > NPImS10/CAL-B (agglomerate size = 37 nm; two reuse cycles).

Support size has been described to have a deep influence in the resulting biomaterial [58], achieving a faster saturation of lipase adsorption with smaller beads [59]. Also, it was observed a 6.3-fold higher catalytic efficiency for pectinase immobilization onto nano-silica support in comparison to the micron-sized silica beads [60]. However, considering that we were working with aggregated particles, this trend might not be observed, since larger aggregates gave more stable immobilized CAL-B than smaller ones (Figure 6), which was also found in Wang et al.’s work [61], but not discussed by the authors. Curiously, in their report, they described the influence of silane chain length on the stability of an immobilized lipase, in which they found out that the larger the chain length, the more stable was the immobilized lipase. Therefore, we suggest that the immobilized CAL-B stability would be influenced by aggregate sizes, glutaraldehyde cross-linking and/or impurity of surfactant. 

In terms of productivity over eight successive cycles, the trend follows NPImS18/CAL-B (337,8% yield) > NPImS10/CAL-B (275,5% yield) > NPImS4/CAL-B (266% yield), which would be in agreement to the FTIR analysis, since NPImS18/CAL-B did not present the 1715 cm^−1^ band, whereas NPImS10/CAL-B was an intermediate between NPImS4/CAL-B and NPImS18/CAL-B systems (Figure 3).

Since the immobilization of CAL-B on NP-ImS10 achieved 45 times more activity than free CAL-B, we employed this system in the transesterification reaction of lipids extracted from the black yeast *Exophiala oligosperma* and from the bacteria *Bacillus subtilis*. The reaction yield with *E. oligosperma* oils were of 89%, whereas the yield using the bacteria oils could not be determined due to its low conversion might be due to the high loads of phospholipids which could act as lipase inhibitors [62]. 

## 3. Materials and Methods

### 3.1. Materials

Lipase from *Candida Antarctica* B (CALB) (powder), imidazole, KOH, K_2_CO_3_, MgSO_4_, FeCl_3_.6H_2_O, FeCl_2_.4H_2_O, NaOH, n-alkylbromide *, 1,3-propane sultone, (3-aminopropyl)triethoxysilane and glutaraldehyde were obtained from Sigma-Aldrich (Saint Louis, Missouri, MO, USA). The solvent solutions (acetonitrile, ethyl acetate, acetone, hexane, dichloromethane, chloroform, methanol, toluene) were purchased from Synth (Diadema, São Paulo, Brazil) and used without previous purification. * Corresponding to n = 4, 10 and 18.

### 3.2. Characterization Techniques 

Nuclear magnetic resonance (^1^H–NMR) analysis were performed using a Bruker 400 MHz spectrometer (Bruker, Fällanden, Switzerland). Scanning electron microscopy (SEM) images were obtained using a FEI Inspect F50 equipment (FEI, Hillsboro, OR, USA). Infrared spectra were recorded on a BOMEM spectrophotometer (ABB BOMEM, Quebec, QC, Canada), MB102 model, using KBr pellets. The X-ray diffraction analyses were carried out on a Bruker Nanostar (Karlsruhe, Germany). Bradford assays were performed using a HP-8452A—Hewlett Packard spectrophotometer (Hewlett Packard, Palo Alto, CA, USA). DLS analysis were performed on a Malvern Zetasizer µV (Malvern, Malvern, UK) using suspensions of 1 g·L^−1^ and 10 g·L^−1^ concentrations, and the obtained sizes were plotted by number of particle size distribution. Microanalysis were performed by Microanalytical Laboratory of Universidade Federal de São Carlos, São Carlos (SP-Brazil), using a Fisions CHN, mod. EA 1108 elemental analyzer (Thermo Scientific, Waltham, MA, USA).

### 3.3. Synthesis of Alkylimidazoles

The zwitterionic surfactants synthesis was performed by adaptation of a literature procedure [22], which consists of two steps: the synthesis of an akyl imidazole molecule followed an alkylation utilizing 1,3-propane sultone.

An imidazole solution (1.30 g, 0.0191 mol) in acetonitrile (10 mL) was added to a round-bottom flask containing potassium hydroxide (0.76 g, 0.013 mol) and potassium carbonate (0.50 g, 0.0036 mol). Then, a solution of n-alkyl bromide (0.012 mol) was added dropwise to the mixture, and the reaction proceeded for 16 h at 80 °C under continuous stirring. After this period, the solids were removed by filtration and the solution was diluted in 100 mL of ethyl acetate and washed three times with 50 mL of distilled water. The organic phase was dried with anhydrous magnesium sulfate, filtrated and the solvent was evaporated under vacuum, giving a yellow residue. The given product was characterized by ^1^H-NMR in CDCl_3_.

### 3.4. Synthesis of ImS-n Sufactants

A solution of 1,3-propane sultone (1.34 g, 0.011 mol) in acetone (12 mL) was slowly added to the flask containing the akyl imidazole (0.012 mol) at 0 °C. The reaction mixture was heated to the room temperature and, then, stirred for 5 days. After that, the reaction solution was filtrated giving the product as a solid. The solid was washed two times with a mixture of 1:1 hexane/dichloromethane and dried under vacuum, giving the zwitterionic surfactant in 51% yield. The given product was characterized by ^1^H-NMR in CDCl_3_.

### 3.5. Magnetic Nanoparticles Synthesis

The magnetic nanoparticles were synthesized by the coprecipitation method previously described [63] using the zwitterionic surfactants for reverse micelle synthesis. A round-bottom flask containing a solution of zwitterionic surfactant (ImS-n) (1.0 g) in chloroform (50 mL) under inert atmosphere was mechanically stirred for 30 min. In parallel, a mixture 1:2 of FeCl_3_·6H_2_O (0.13 g, 4.81 × 10^−4^ mol) and FeCl_2_·4H_2_O (0.0062 g, 3.12 × 10^−5^ mol) was added to deoxygenated distilled water (4 mL). The solution containing iron ions was added dropwise to the surfactant solution under stirring, giving a colloidal suspension of reverse micelles. Finally, an aqueous solution of sodium hydroxide (0.0038 mol, 1 mL) was quickly added to the suspension. The reaction was allowed to proceed for 30 min at room temperature. Subsequently, the black powder (Fe_3_O_4_) was magnetically separated and washed with deoxygenated water until pH 7. The same procedure was applied using the three ImS-n molecules, obtaining three different sizes of nanoparticles (NP-ImS4, NP-ImS10 and NP-ImS18).

### 3.6. Nanoparticles Functionalization with Silanol Groups 

The magnetic nanoparticles were washed two times with deoxygenated methanol and dispersed in 50 mL of a mixture 1:1 of methanol/toluene under inert atmosphere. Then, (3-aminopropyl) triethoxysilane (1 mL, 0.0043 mol) was added to the mixture and the system refluxed for 12 h at 110 °C. The solvent was removed by evaporation under vacuum, the black powder (Fe_3_O_4_@APTES) was washed with distilled water and dried under vacuum. The obtained particles were characterized by scanning electron microscope (SEM), infrared spectroscopy (IR), dynamic light scattering (DLS), X-Ray diffraction Powder (XRD) and microanalysis. CHN for NP-ImS_4_ (3.16/1.70/0.90), NP-ImS_10_ (5.56/1.64/2.04) and NP-ImS_18_ (17.12/4.23/5.83).

### 3.7. Immobilization of Lipase B from *Candida antarctica* on Fe_3_O_4_@APTES

For lipase immobilization, a modified literature procedure [64] was employed. A small amount (0.01 g) of silanized nanoparticles (Fe_3_O_4_@APTES) were dispersed in a phosphate buffer solution pH 7 (0.8 mL). Then, 10 μL of a glutaraldehyde solution (2.76 mol × L^−1^) was added, followed by 200 μL of a lipase solution (10 mg/mL, 300 μmol × L^−1^). The mixture was stirred for 1 h at room temperature. The black powder (Fe_3_O_4_@APTES-CALB) was magnetically separated, washed with distilled water and dried. The enzyme load was determined by Bradford method, quantifying the difference in absorbance before and after the immobilization procedures. 

### 3.8. Lipase Catalytic Activity Optimization

Catalytic activity optimization experiments were carried out using soybean oil (0.5 mL), methanol (60 μL) and the biocatalyst Fe_3_O_4_@APTES-CALB. Three parameters were evaluated during the immobilization process: amount of the nanoparticles (3, 5, 7 and 10 mg), glutaraldehyde concentration (0.028 mol × L^−1^, 0.055 mol × L^−1^, 0.083 mol x L^−1^ and 0.110 mol × L^−1^) and immobilization time (30, 60, 120, 180 and 240 min.). After the immobilization, immobilized CAL-B was subjected to a transesterification reaction using soy bean oil and methanol without using any additional solvents. The total content of Fe_3_O_4_@APTES-CALB was added to 250 μL of soy bean oil with 20 μL of methanol, then, 2 equivalents of methanol was added in three steps (after 150 and 300 min of the reaction) and the system was stirred at 37 °C for 24 h. After that period, the nanoparticles were magnetically separated, washed with hexane and dried. The reaction product was characterized by ^1^H-NMR, quantifying FAMEs production.

The same procedure was carried out using the three different sizes of magnetic nanoparticles obtained thought the application of the ImS-4, ImS-10 and ImS-18 surfactants molecules.

### 3.9. Microbial Lipid Extraction Process 

Total lipids from *B. subtilis* were extracted using 1 L of cultures in LB medium with optical density of ≈1, employing the Bligh and Dyer method [65].

Total lipids from *E. oligosperma* were obtained after placing the yeast extract in an Erlenmeyer flask with 500 mL of ethyl acetate under ultrasonic bath for one hour (3×). Then, the mixture was filtrated in a Buchner funnel, dried with anhydrous magnesium sulfate (MgSO_4_), and the solvent was evaporated under vacuum. The given substrate was fractioned using silica column chromatography applying a solvent mixture of hexane/ethylacetate 1:1, achieving 0.315 g of a yellowish oil. 

### 3.10. Biodiesel Synthesis Catalyzed by Lipase B from *Candida antarctica* Immobilized on Magnetic Nanoparticles

The transesterification reaction was carried out using a lipid fungus extraction and methanol without using any additional solvents. A little amount of Fe_3_O_4_@APTES-CALB (10 mg) was added to 250 μL of extracted lipid (containing triacylglyerols) with 20 μL of methanol, then, 2 equivalents of methanol was added in three steps (after 150 and 300 min of the reaction) and the system was stirred at 37 °C for 24 h. After that, the nanoparticles were magnetically separated, washed with hexane and dried. The reaction product was characterized by ^1^H-NMR.

### 3.11. Determination of the Transesterification Reaction Activation Energy 

The transesterification reaction activation energy was also determined using soybean oil (0.5 mL), methanol (60 μL) and the biocatalyst Fe_3_O_4_@APTES-CALB (10 mg). The system was stirred at three different temperatures (27 °C, 32°C and 37 °C) and every two hours 20 μL of the reaction mixture were taken out, dried in the vacuum and characterized by ^1^H-NMR. The same procedure was carried out using the three different sizes magnetic nanoparticles obtained thought the application of the ImS-4, ImS-10 and ImS-18 surfactants molecules and using the free lipase for comparison. Constant *k* was determined by the reaction yields at different temperatures, using an adapted method from O´Connor et al. [66]. An Ahrrenius plot (k vs. 1/T(K)) gave the results of energy of activation, whereas the entropy of activation was calculated using the Eyring equation [67].

## 4. Conclusions

Lipase B from *Candida antarctica* was used as a model enzyme to study the effects of immobilization on the structure, activity, and stability. Three different magnetic nanoparticles were synthesized by the coprecipitation method using zwitterionic surfactants. It was seen that although the same conditions were used in the immobilization and catalytic procedure, infrared analysis showed that NP-ImS4 and NP-ImS10 cross-linked to CAL-B via a Michael-type addition, which generated less stable immobilized CAL-B when compared to NP-ImS18, which was bonded to the enzyme via a pyridine formation. The highest stability observed for NP-ImS18/CAL-B system might also be associated to the initial size of particle agglomerates, since the larger agglomerate size, the higher immobilized CAL-B stability was observed. In relation to CAL-B activity, transesterification reactions of soy bean reached up to 90% of conversion into biodiesel using the immobilized CAL-B, whereas the free enzyme only achieved a yield of 2% due to its denaturation/unfolding process in the presence of high methanol concentrations. Finally, the transesterification reaction of lipids extracted from a fungi achieved a yield of 89%, confirming the potential use of immobilized CAL-B in biodiesel production from renewable oil sources.

## Figures and Tables

**Table 1:** Thermodynamic and kinetic parameters of the activation energy and entropy associated to the transesterification reaction yield for free and immobilized CAL-B.

Biocatalyst	E_a_ (kJ mol^−1^)	ΔS ^≠^ (J mol^−1^)	*k* (μMs^−1^) ^a^	Yield (%) ^b^
Free CAL-B	29.4	−73	0.048	2/1.5 ^c^
NP-ImS4/CAL-B	25.6	−138	33.2	79.7
NP-ImS10/CAL-B	18.2	−142	21.7	90
NP-ImS18/CAL-B	27.8	−145	14.0	83

a- Determined at 32 °C; b- determined at at 32 °C and 24 hours.; c- reaction in presence of ImS10 surfactant.

## Supplementary Materials

The Supplementary materials are available online.
