# Supplementary material for: Influence of Glutaraldehyde Cross-Linking Modes on the Recyclability of Immobilized Lipase B from Candida antarctica for Transesterification of Soy Bean Oil
Source: Molecules. 2018 Sep 2;23(9):2230. doi: 10.3390/molecules23092230 (PMC6225267; doi:10.3390/molecules23092230)
Supplement: Supplementary file 1 [file molecules-23-02230-s001.pdf]

Influence of glutaraldehyde cross-linking modes on the recyclability of immobilized lipase B from *Candida antarctica* for the transesterification of soy bean oil

Modenez, I. A.[1], Sastre, D. E.[2], Moraes, C. F. [3], Marques Netto, C.G.C.*[1]

1- Laboratório de Metaloenzimas e Biomiméticos, Departamento de Química, UFSCar, São Carlos-SP, Brazil *email: caterina@ufscar.br
2- Departamento de Física e Ciências Interdisciplinares, Instituto de Física, Universidade de São Paulo, São Carlos-SP, Brazil
3- Laboratório de Analítica, Bioanalítica, Biossensores, Eletroanalítica e Sensores, Departamento de Química, UFSCar, São Carlos-SP, Brazil

S1. 1H NMR spectra of ImS4 zwitterionic surfactant. 2

S2. ^{1}H NMR spectra of ImS10 zwitterionic surfactant. 3

S3. ^{1}H NMR spectra of ImS18 zwitterionic surfactant. 3

S4. DRX of NP-ImS4. 4

S5. DRX of NP-ImS10. 4

S7. SEM of NP-ImS4. 5

S8. SEM of NP-ImS10. 6

S9. SEM of NP-ImS18. 7

S10. Number of particle size distribution of NP-ImS4 at 1gL^{-1} concentration. 8

S11. Number of particle size distribution of NP-ImS10 at 1gL^{-1} concentration 8

S12. Number of particle size distribution of NP-ImS18 at 1gL^{-1} concentration 9

S13. Number of particle size distribution of NP-ImSn (n=4, 10 and 18) at 10gL^{-1} concentration. 10

S16. ^{1}H NMR spectra of oleyltriacylgliceride. 12

S17. FTIR spectra of immobilized CAL-B on NP-ImS18 at pHs 4 (black line), 7 (red line) and 11 (blue line). 12

S18. FTIR spectra of immobilized CAL-B on NP-ImS4 in a system without ImS18 as a contaminant (black line) and with ImS18 as a contaminant (red line) 13

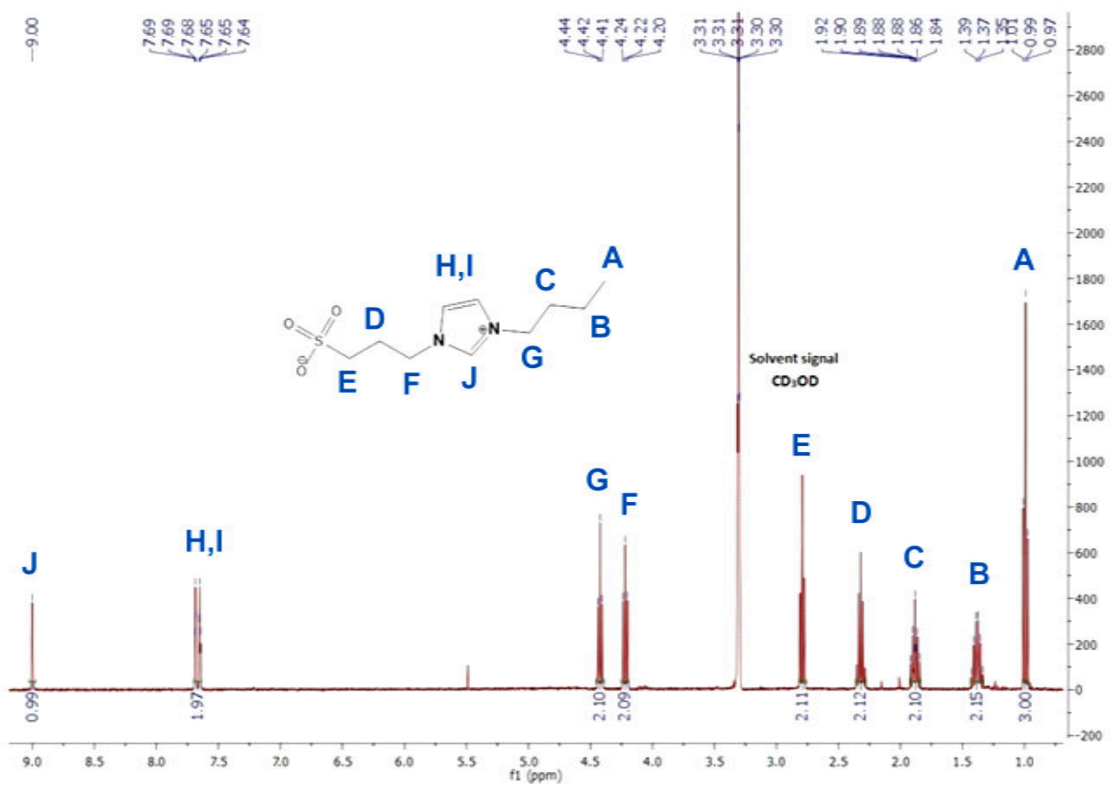

S1. 1H NMR spectra of ImS4 zwitterionic surfactant.

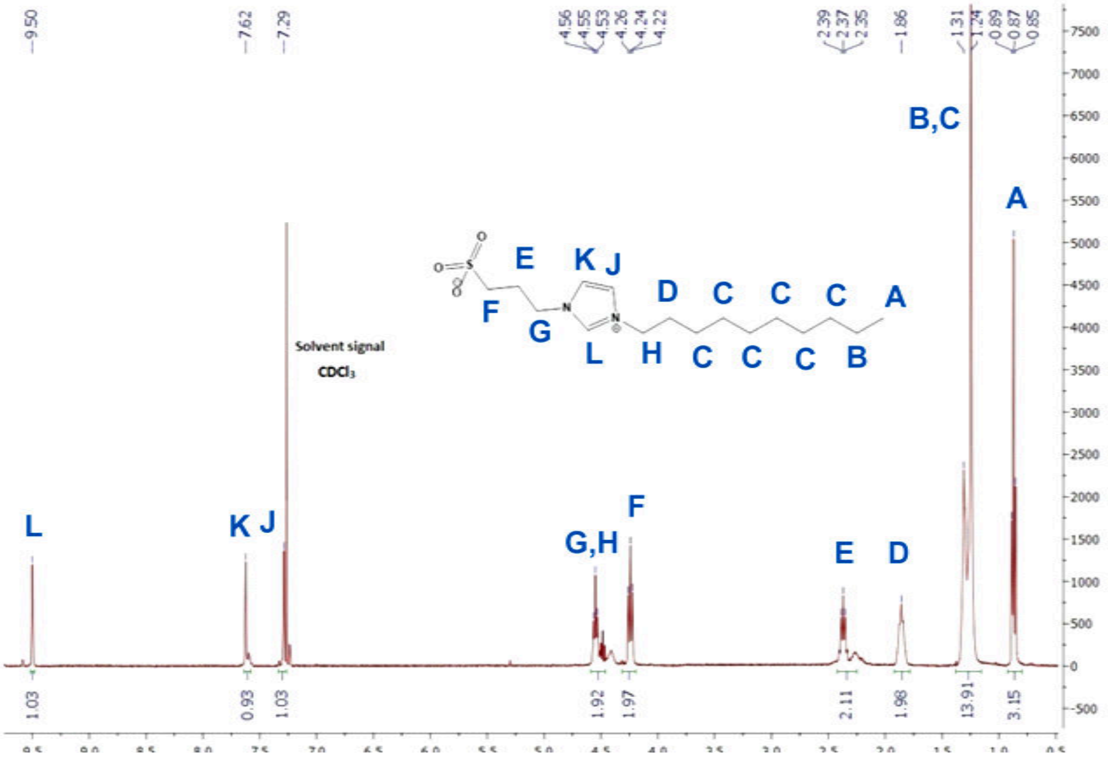

S2. ^{1}H NMR spectra of ImS10 zwitterionic surfactant.

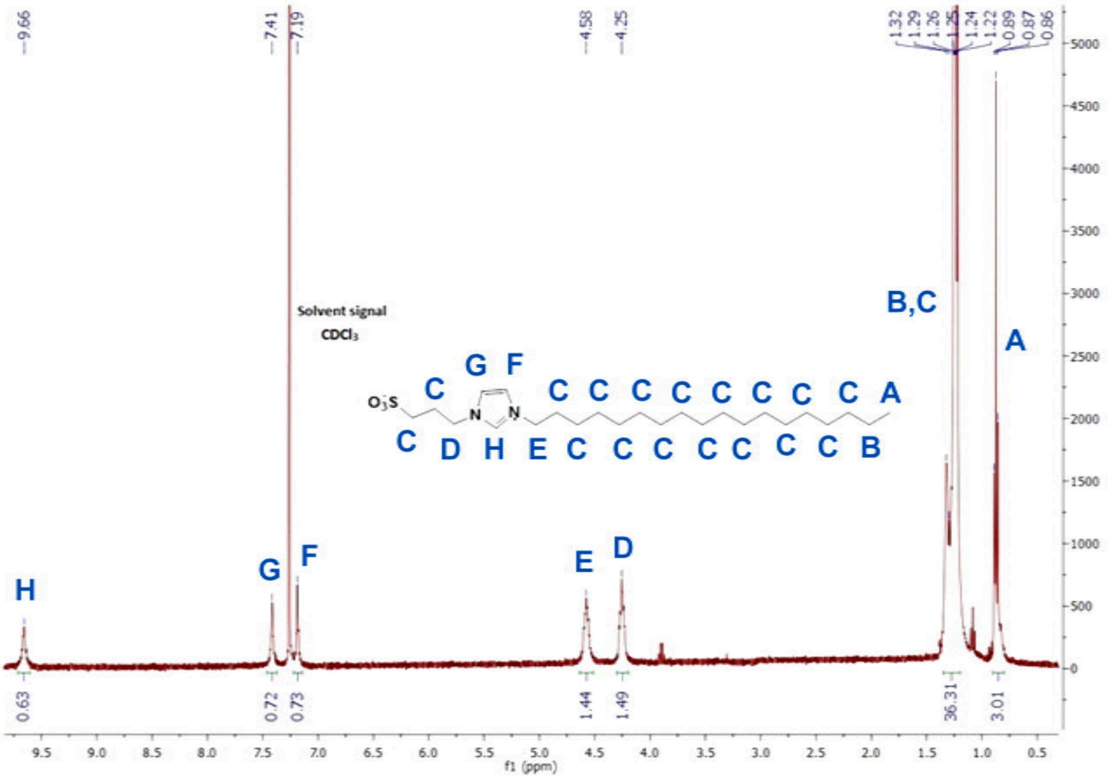

S3. ^{1}H NMR spectra of ImS18 zwitterionic surfactant.

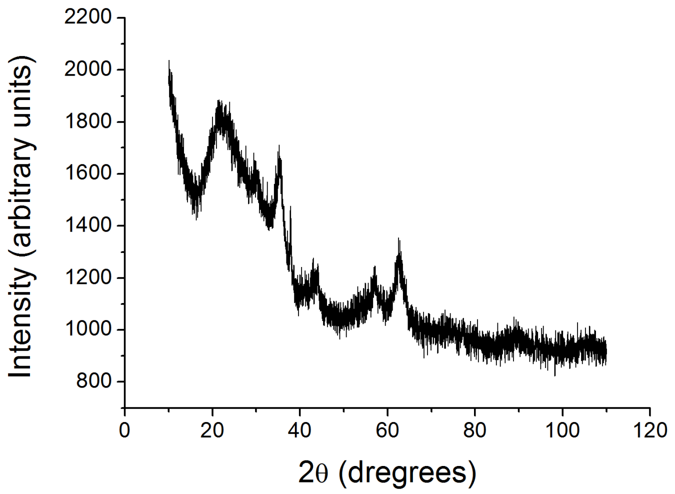

S4. DRX of NP-ImS4.

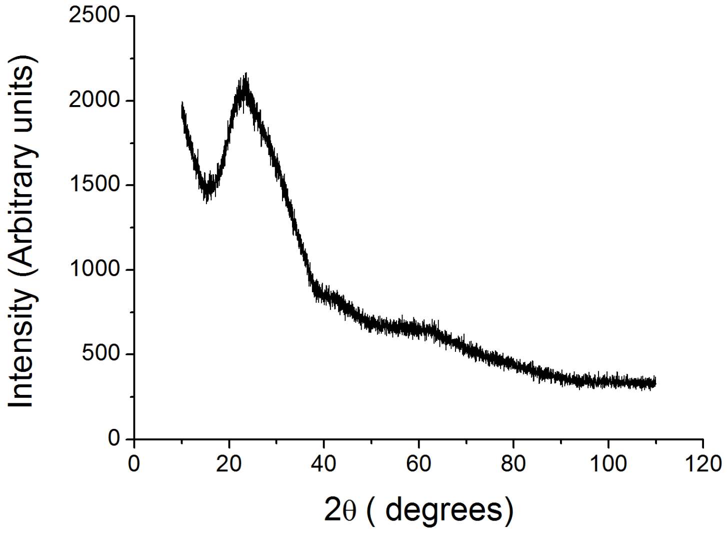

S5. DRX of NP-ImS10.

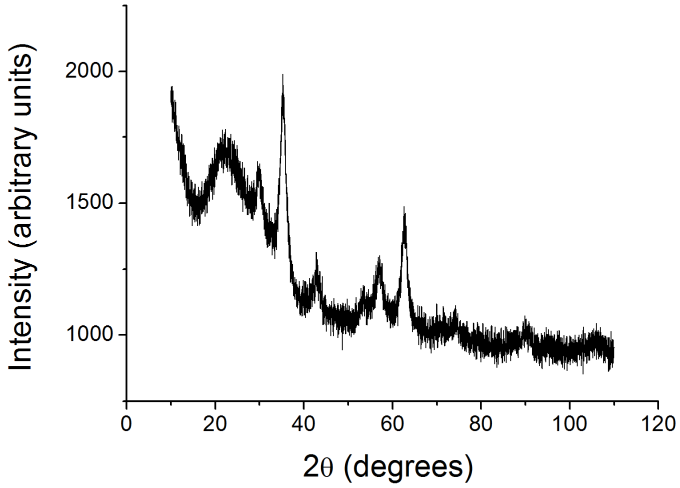

S6. DRX of NP-ImS18.

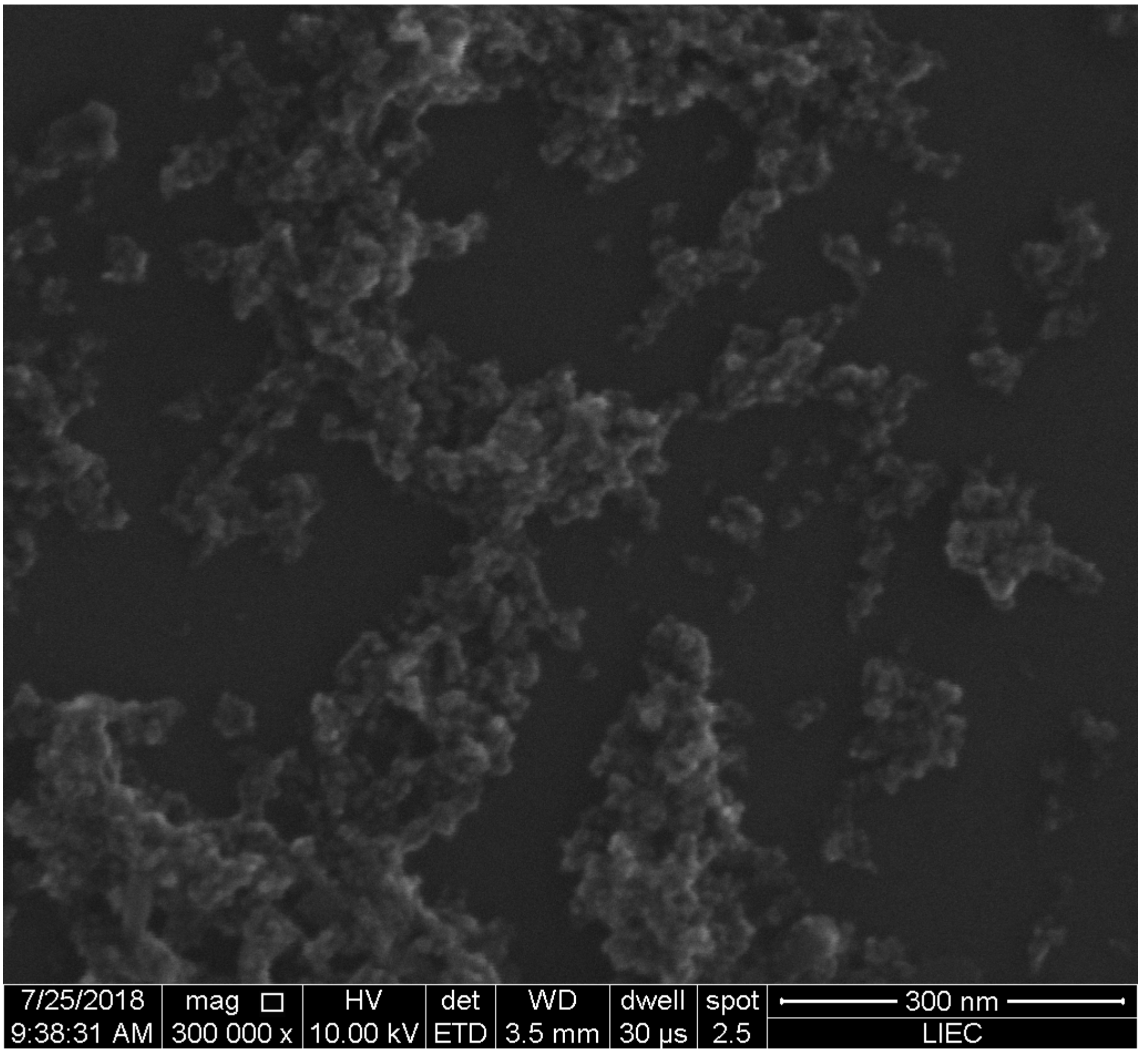

S7. SEM of NP-ImS4.

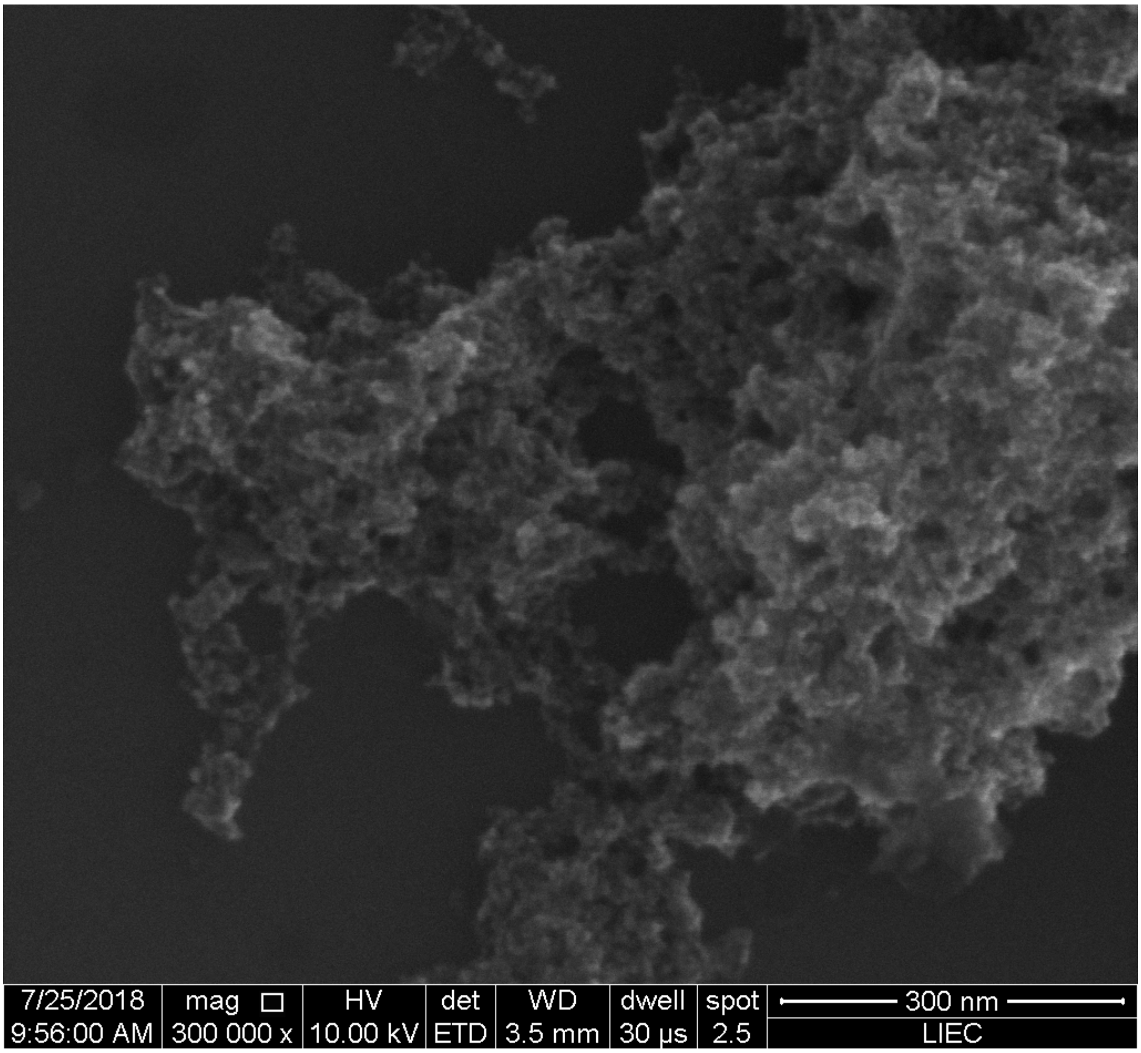

S8. SEM of NP-ImS10.

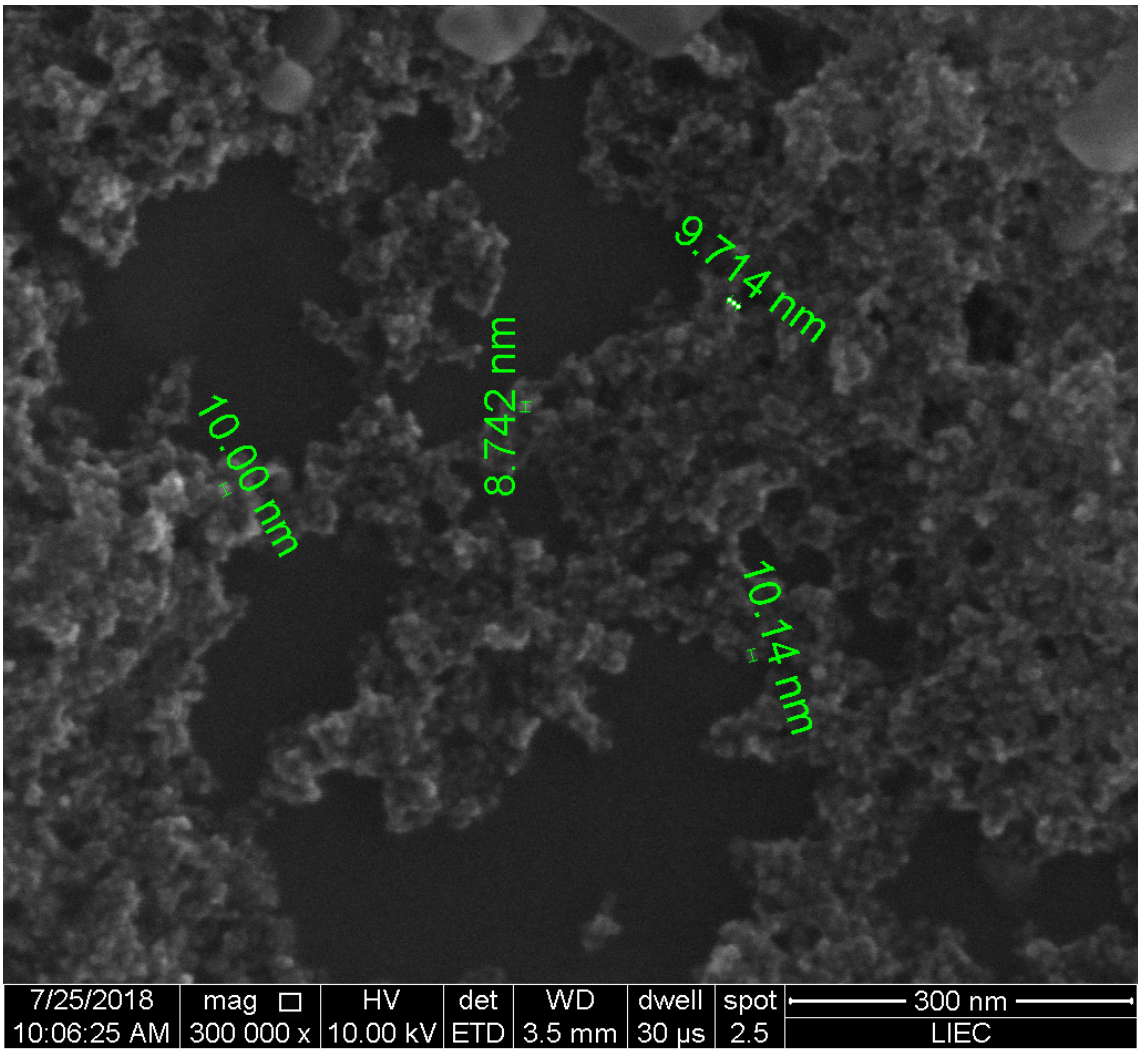

S9. SEM of NP-ImS18.

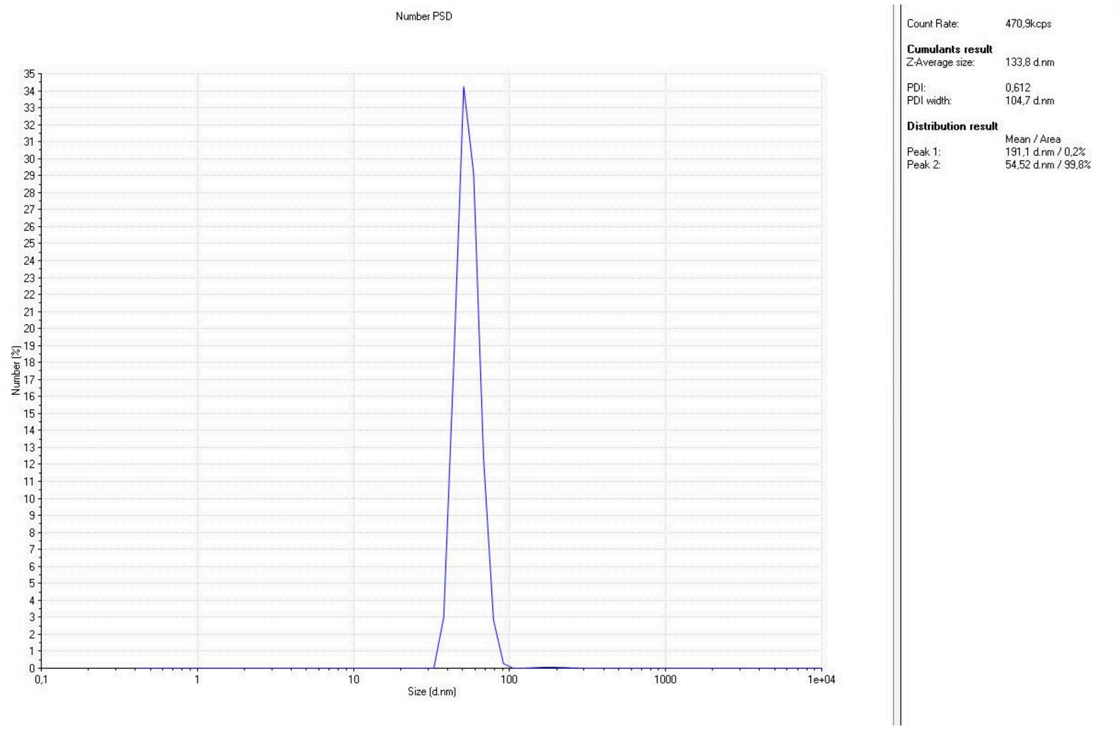

S10. Number of particle size distribution of NP-ImS4 at 1gL^{-1} concentration.

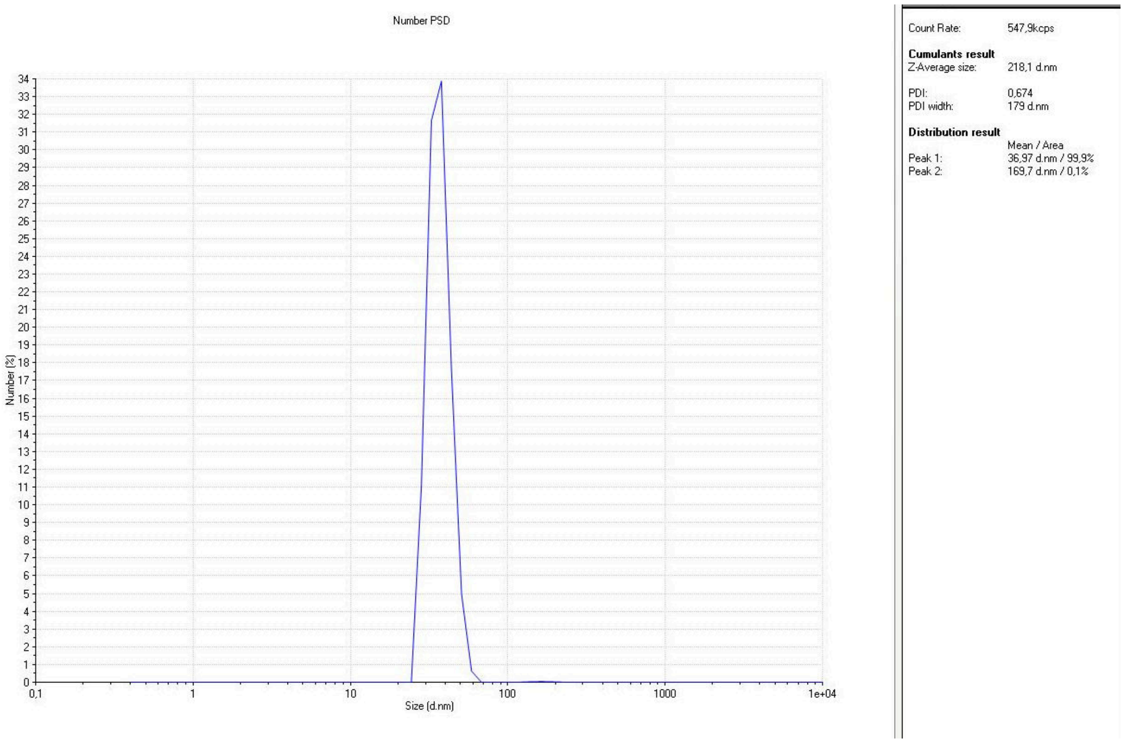

S11. Number of particle size distribution of NP-ImS10 at 1gL^{-1} concentration

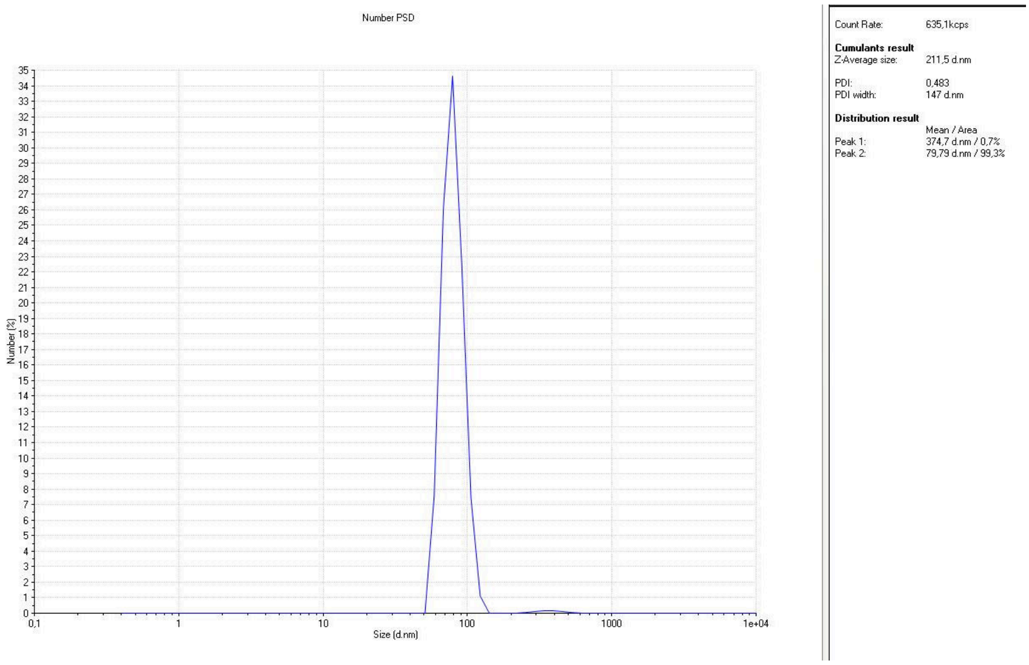

S12. Number of particle size distribution of NP-ImS18 at $1 gL^{-1}$ concentration

NP-ImS4

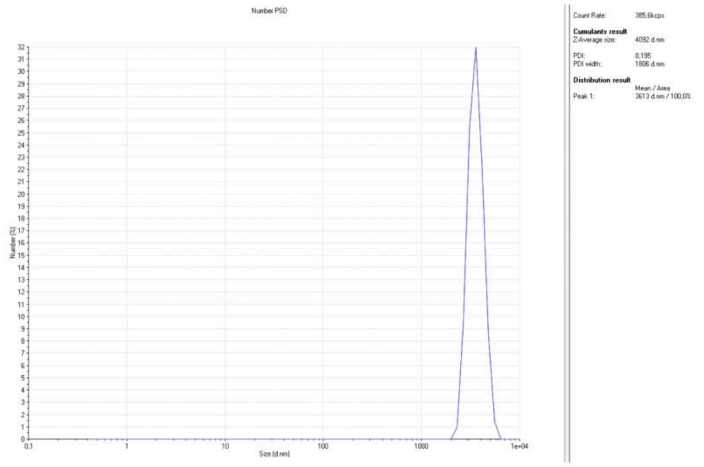

NP-ImS10

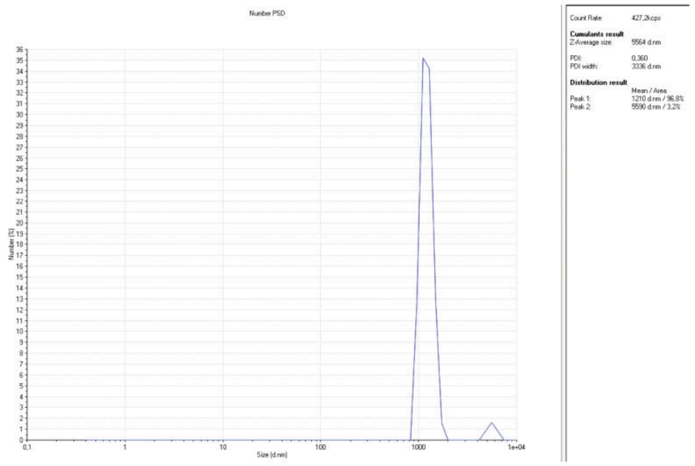

NP-ImS18

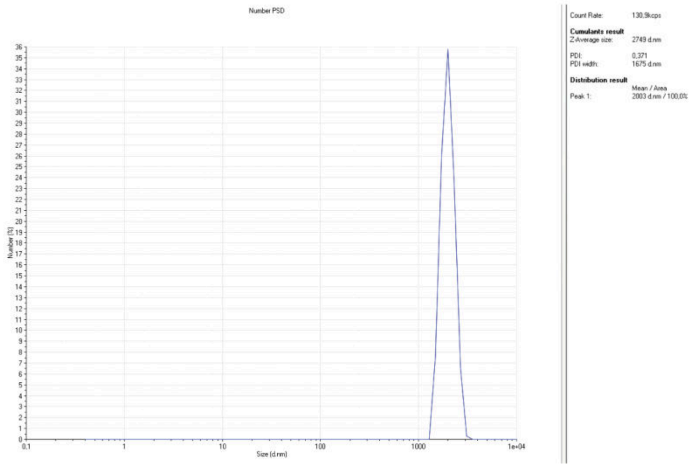

S13. Number of particle size distribution of NP-ImSn (n=4, 10 and 18) at 10gL^{-1} concentration.

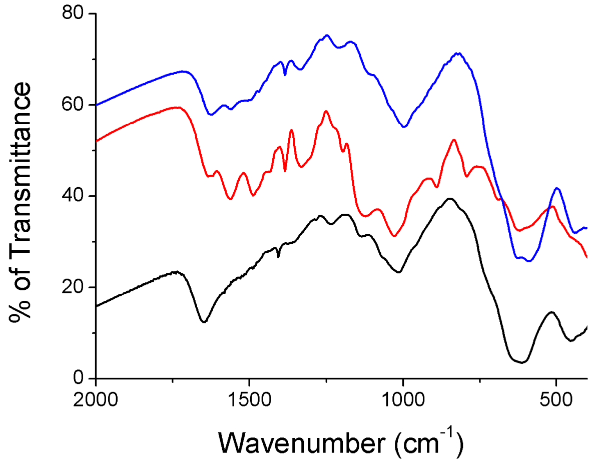

S14. Infrared of silanized magnetic nanoparticles. NP-ImS4 (black line); NP-ImS10 (red line) and NP-ImS18 (blue line).

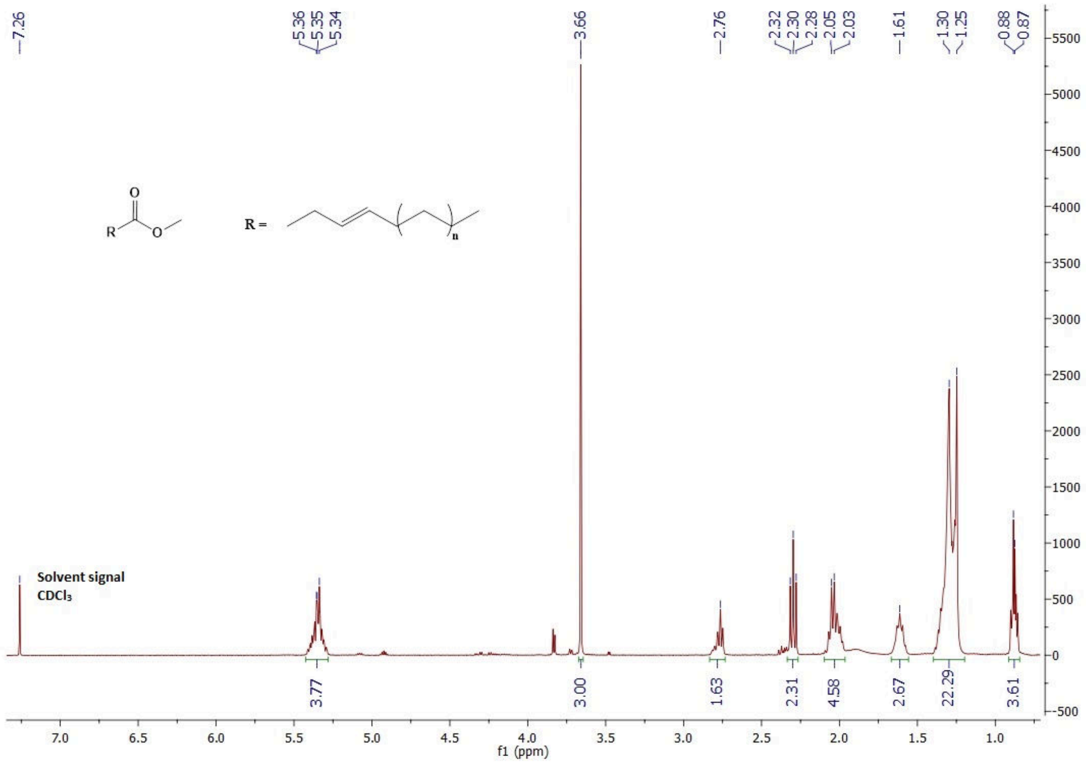

S15. 1H NMR spectra of methylated fatty acids.

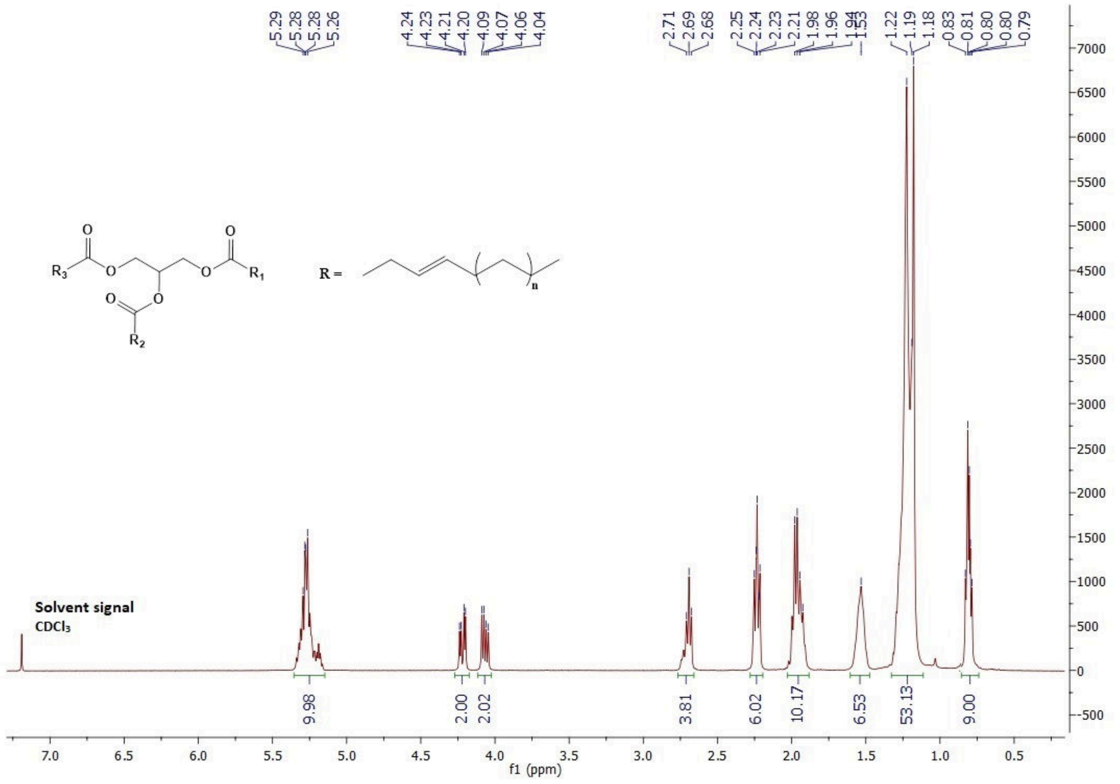

S16. ^{1}H NMR spectra of oleyltriacylgliceride.

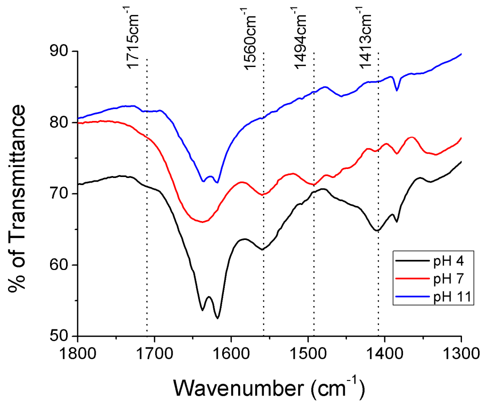

S17. FTIR spectra of immobilized CAL-B on NP-ImS18 at pHs 4 (black line), 7 (red line) and 11 (blue line).

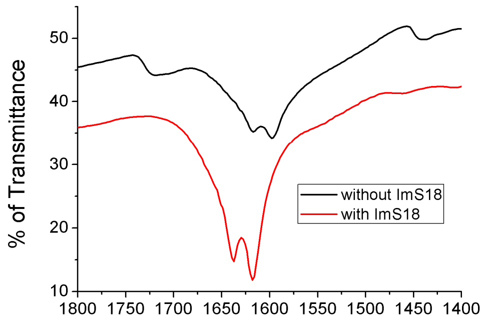

S18. FTIR spectra of immobilized CAL-B on NP-ImS4 in a system without ImS18 as a contaminant (black line) and with ImS18 as a contaminant (red line) .
